# Is gene therapy a good therapeutic approach for HIV-positive patients?

**DOI:** 10.1186/1479-0556-5-5

**Published:** 2007-02-14

**Authors:** Jai G Marathe, Dawn P Wooley

**Affiliations:** 1 Department of Neuroscience, Cell Biology, and Physiology, Wright State University, Dayton, OH 45435, USA; 2 Center for Retrovirus Research, The Ohio State University, Columbus, OH 43210, USA

## Abstract

Despite advances and options available in gene therapy for HIV-1 infection, its application in the clinical setting has been challenging. Although published data from HIV-1 clinical trials show safety and proof of principle for gene therapy, positive clinical outcomes for infected patients have yet to be demonstrated. The cause for this slow progress may arise from the fact that HIV is a complex multi-organ system infection. There is uncertainty regarding the types of cells to target by gene therapy and there are issues regarding insufficient transduction of cells and long-term expression. This paper discusses state-of-the-art molecular approaches against HIV-1 and the application of these treatments in current and ongoing clinical trials.

## Background

In 1983, a new virus was first isolated and associated with acquired immune deficiency syndrome (AIDS) [1]. Subsequently, scientists classified it as a *Lentivirus *belonging to the family *Retroviridae *and named it human immunodeficiency virus (HIV) [2]. HIV infection not only causes physical debility but also has negative social implications [3-7]. During the later stages of HIV infection, patients develop AIDS, presenting with severely depleted CD4^+ ^T-cell counts (<200 cells per microliter of blood) along with a myriad of opportunistic infections. According to the Joint United Nations Programme on HIV/AIDS, approximately 30 million people have lost their lives since the identification of the first AIDS patients in 1980. The global number of HIV-positive patients is around 39.5 million as of December 2006. There was an estimated average of 2.9 million deaths and 4.3 million new cases in 2006 [8].

### Why consider gene therapy as a treatment modality?

Despite thousands of researchers worldwide working on a cure for HIV infection, none of the modalities have been completely successful. Currently, four classes of anti-retroviral drugs are available: nucleoside/nucleotide analogs, non-nucleoside reverse transcriptase inhibitors, protease inhibitors, and fusion (or entry) inhibitors. These drugs, used in various combinations to treat HIV, form what is known as highly active antiretroviral therapy (HAART). However, HAART is expensive, has high toxicity rates, and must be administered lifelong, i.e. it is not curative. In addition to the above problems, the rate of emergence of resistant strains is high post-HAART. In studies conducted in the United States and Europe, over 50% of patients experienced virologic failure (viremia) while on antiretroviral therapy, and approximately 80% of these patients showed drug resistant HIV genotypes [9,10]. One long-term study found that by six years, approximately 80% of patients had their medications switched repeatedly due to drug resistance, resulting in an overall cumulative failure rate of 38% [11], placing these patients in danger of exhausting their treatment options [12]. Transmission of drug resistant HIV mutants is also an increasing problem. In a study among newly infected individuals, 14% of patients were infected with HIV that already had one or more key drug resistance mutations [13]. For these reasons, there is an increasing urgency to find a cure for HIV infection.

With the advent of the molecular and genetic age of medicine, research to create gene therapy for HIV has been on the rise. Since the 1980's, researchers have explored the possibility of using gene therapy to cure HIV-positive patients. In 1988, David Baltimore used the term 'intracellular immunization' to describe this treatment approach [14]. Initial *in vitro *experiments were successful and now scientists are applying some of these methods in clinical trials.

### Strategies for inhibiting HIV

**Figure 1:**
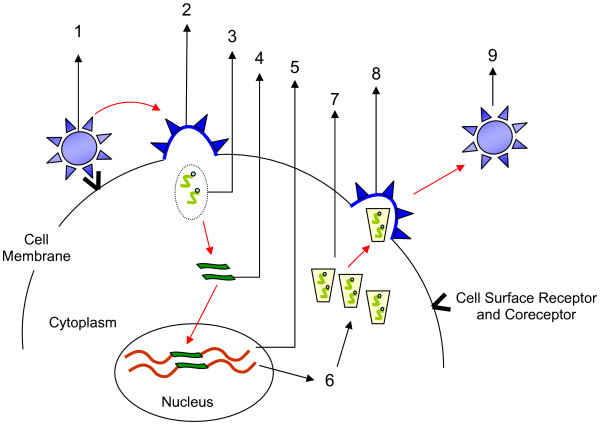
Schematic representation of the life cycle of HIV and the various steps at which anti- HIV gene therapy could be applied with key viral target proteins in parentheses: (1) HIV-1 attachment and binding (Env, gp120); (2) HIV-1 entry (Env, gp41); (3) Reverse transcription (reverse transcriptase and Vif); (4) Transport of HIV-1 DNA into the nucleus and integration with cellular DNA (Vpr, matrix, integrase). (5) Transcription of the HIV-1 proviral genome to produce both spliced and unspliced HIV-1 RNAs (Tat); (6) Transport of HIV-1 transcripts to cytoplasm (Rev); (7) HIV-1 gene expression and posttranslational modification of HIV-1 proteins (Gag, Gag-Pol, and Env polyproteins, Vif, and Nef). (8) HIV-1 virion assembly and morphogenesis within the cell (all virion proteins). (9) Release and maturation of the immature virion into a completely infectious particle (protease, Vpu, and Nef).

Figure 1 is a schematic representation of the life cycle of HIV showing the various stages at which genetic therapy could be applied. Therapy could also be aimed at any one of the many target cells for HIV infection *in vivo*, including immune cells such as CD4^+ ^and CD8^+ ^T cells, dendritic cells, monocytes, macrophages, hematopoietic stem cells (HSCs), brain cells, and other cells from the gastrointestinal tracts that could serve as host cells for HIV. Since T cells are the major cell population implicated in HIV infection and its progression to AIDS, making these cells immune to infection is a very important aspect of therapy. Even more desirable are the HSCs. These self-replicating progenitor cells give rise to all other members of the lymphoid and myeloid lineages and have the capability of repopulating the immune system with a potentially HIV-resistant phenotype.

A variety of viral or cellular components could serve as targets for anti-HIV gene therapy. Targeting viral factors is currently the most prevalent method. A major problem with this strategy is that HIV can quickly form resistant strains to these genetic modifications due to high mutation rates. Targeting cellular factors makes the occurrence of resistant strains less likely but raises the issues of adverse effects on normal cell function. HIV receptors and co-receptors are attractive targets, but there have been recent reports of liver toxicity with CCR5 antagonists in HIV-infected patients [15]. A summary of the main anti-viral approaches is as follows:

### Protein-based strategies

#### A. Introduction of suicide genes

Cells modified with suicide genes for negative selection. Generally, suicide genes code for enzymes that convert an inactive drug to a toxic form, allowing for the potential killing of the modified cells. The idea of using suicide genes was made popular as a potential approach for treating cancer. As an anti-HIV strategy this method was tried *in vitro *as proof-of-concept in a study that used a retrovirus to transduce autologous CD8+ cells from HIV-infected patients; the suicide gene was thymidine kinase expressed as a fusion protein with hygromycin phosphotransferase [16]. As with other gene therapy approaches, specific targeting of desired HIV-infected cell populations would present a challenge for this approach to be successful *in vivo*.

#### B. Transdominant negative proteins

Mutations in a specific gene expressed in a dominant fashion where the mutant protein can interfere with the function normally carried out by the parent gene product. If the protein is multimeric, the nonfunctional protein can multimerize with the normal protein and the resultant complex is functionally inactive. Thus, the dominant negative protein can have a strong inhibitory effect on the normal protein formation or function. Examples of this are HIV-1 Gag mutants [17] and Rev M10 [18]. Rev M10 was the first transdominant negative protein used in clinical trials; it is a dominant negative mutant capable of binding the Rev Responsive Element (RRE) and has the capability of forming multimers [18].

#### C. Chimeric receptors

CD4/CD3 chimeric receptor called CD4ζ. The CD4ζ has the extracellular and transmembrane domains of the CD4 antigen and the intracytoplasmic domain of the CD3 T cell receptor. The extracellular portion binds HIV, while the cytoplasmic portion initiates a signaling cascade similar to the one initiated by the normal T-cell receptor (TCR) binding with HIV [19]. Thus, engagement of CD4ζ with HIV results in the generation of an HIV-specific T cell response. CD4ζ-modified T lymphocytes inhibit viral replication in T cells and macrophages *in vitro *[20] and mediate killing of HIV-infected T cells [19].

#### D. Intracellular HIV-1 specific single chain antibodies (SFv)

These antibodies bind to viral proteins intracellularly and block their action, e.g. anti-Rev SFv [21] and anti-gp120 SFv [22].

### RNA-based strategies

#### A. RNA decoys

Small RNA molecules containing essential *cis*-acting elements that bind *trans*-acting proteins. They function by luring away *trans*-acting proteins from their true target sequence. When expressed at high levels, they can successfully compete against viral *cis*-acting sequences that are indispensable for viral replication [23,24].

#### B. Ribozymes

Small RNA molecules also called 'catalytic RNAs'. They cleave the target RNA at specific sequences. The binding arms of the ribozyme molecule determine the specific cleavage sites. Ribozymes can target critical sites such as *gag *[25], *tat *[26], and U5 [27].

#### C. Antisense RNA

Antisense RNA is single-stranded RNA bearing complementary nucleotide sequences to a target RNA. It pairs with the target RNA forming a double-stranded RNA structure that either blocks translation or becomes a target for degradation in the cell. Attractive targets for this type of therapy are *gag *and other conserved regions of HIV [28].

#### D. RNA interference (RNAi)

Small interfering RNAs (siRNA) are double-stranded RNA sequences, approximately 22 base pairs in length that bind cellular mRNAs in a sequence-specific manner and cleave them at the center of the complementary region. They achieve this through a series of steps involving the recruitment of RNAi factors, formation of an RNA-induced silencing complex (RISC), unwinding of the siRNA, and activation of RISC [29]. Short hairpins RNAs (shRNAs) can also induce gene silencing [30]; a ribonuclease III type protein called Dicer cuts off the loop of the hairpin to form an intracellular siRNA. To date, small interfering RNAs have been used *in vitro *to target viral genes like *tat *and *rev *[31] and cellular genes like CCR5 [32] with great success.

### Protein and nucleic acid-based strategies

#### Aptamers

In general, aptamers refer to short RNA, DNA, or protein sequences that bind a variety of specific target molecules, including nucleic acids and proteins. Small RNA aptamers have shown success *in vitro *against HIV Rev [33,34] and reverse transcriptase [35]. Aptamers can be used in conjunction with other therapies, such as ribozymes [35].

### Application of bench-side treatment strategies to bedside

The early success of *in vitro *studies paved the way for clinical trials. All the above-mentioned strategies for gene therapy have shown good anti-HIV activity *in vitro*. However, not all of them have been tested in clinical trials. As reported in the literature, the trials conducted to date have used a suicide gene (HyTK), transdominant negative proteins (Rev M10, TdRev, or huM10), a chimeric receptor (CDζ), an RNA decoy (RRE), and ribozymes (anti-*tat*/*vpr *ribozyme and *tat *ribozyme, RRz2). A trial using siRNA was started recently and the results have not yet been published in literature. Following is an account of these clinical trials and their results (see Table 1 for summary).

**Table 1:** Summary of results of clinical trials

Target cells	Vector	Transgene	Anti-HIV method	Results
CD8^+^	Retrovirus	HyTk	Introduction of suicide gene	CTL response cleared modified cells [16].
CD4^+^	Gold-particle-mediated	Rev M10	Transdominant negative protein	Detected Rev M10 until 2 months post infusion, preferential survival [36].
CD4^+^	Retrovirus	Rev M10	Transdominant negative protein	Detected Rev M10 until 6 months post infusion, preferential survival [37].
CD4^+^	Retrovirus	TdRev and/or anti-sense TAR	Transdominant negative proteins and anti-sense RNA	Anti-HIV genes consistently detected for >100 weeks in six of six patients. Preferential survival of transduced cells during a period of high viral load in one patient [47].
CD34^+^	Retrovirus	TdRev	Transdominant negative protein	One patient died due to relapse to Hodgkin's disease. In second patient, detected vector in the progeny for >3 years, remission of leukemia and good viral load control achieved by administering HAART that cannot be attributed to gene therapy [38,39].
CD34^+^	Retrovirus	huM10	Transdominant negative protein	huM10 could be detected in peripheral blood mononuclear cells (PBMC) for 1–3 months and then dropped to at or below the limit of detection over a two year follow-up period. Preferential survival of transduced cells during a period of high viral load in one patient [40].
CD4^+^	Retrovirus	CD4ζ	Chimeric receptor	Decrease of greater than 0.5 log mean in rectal tissue-associated HIV RNA for at least 14 days, detected CD4ζ in 1–3% of PBMCs at 8 weeks [42]
CD4^+^	Retrovirus	CD4ζ	Chimeric receptor	Good expression of CD4ζ for at least 24 weeks in all patients; no difference between control and study group [43].
CD4^+ ^and CD8^+^	Retrovirus	CD4ζ	Chimeric receptor	In 11 of 12 patients who received higher doses of modified CD8^+ ^cells (10^9 ^or 10^10^), CD4ζ could be detected post-infusion for at least 15–40 weeks when they received additional infusions of modified cells. The group receiving IL-2 along with modified CD8^+ ^cells showed a higher persistence of CD4ζ as compared to the group receiving no IL-2. In patients who received modified CD8^+ ^and CD4^+ ^cells, the cells were detected in the peripheral blood for at least 1 year post-infusion [41].
CD34^+^	Retrovirus	(RRE) decoy	RNA decoy	RRE-decoy-containing leukocytes could be isolated from peripheral blood even 1 year post-infusion but the numbers were extremely low [44].
CD4^+^	Retrovirus	RRz2	Ribozyme	Over a 4 year period, PBMCs containing both RRz2 and LNL6 were consistently detected [46].
CD34^+^	Retrovirus	*tat*/*vpr *ribozyme	Ribozyme	Vector was detected in naïve T cells for >3 years; no correlation between changes in viremia or CD4+ T cell counts with vector expression or its detection in any cell type [45].

### Protein-based approaches used in the clinic

In 1996, Riddell *et al*. published one of the earliest trials, involving the use of a suicide gene [16]. This study enrolled six HIV-seropositive patients. Autologous CD8^+ ^T cells were genetically modified using a retrovirus-mediated gene transfer technique. The retrovirus, designated HyTK, comprised the hygromycin phosphotransferase gene (Hy) and the herpes virus thymidine kinase gene (TK) as a fusion gene under the control of the murine leukemia virus (MLV) long terminal repeat (LTR). Hygromycin was used to positively select for transduced autologous cells *in vitro *prior to infusion into the patient. Ganciclovir could have been used, if necessary, to negatively select (kill) the transduced cells in the patient. In four increasing doses, researchers transfused autologous HyTK-transduced CD8^+ ^cells at 14-day intervals. There were no significant side effects. However, five of six patients developed a CTL response to the foreign protein and thus rejected the modified CD8^+ ^cells, which cleared in response to each subsequent transfusion. The results of this trial suggested other strategies that would make modified cells less susceptible to the immune response and thus inspired further research [16].

In the same year, Woffendin *et al*. published the results of a pilot trial involving a transdominant negative protein approach [36]. For genetic modification of CD4^+ ^T cells, they used Rev M10 and a deletion mutant of Rev M10, which showed no antiviral activity. Woffendin and colleagues transduced the T cells using a non-viral vector and showed that following transfusion, there was a preferential survival of Rev M10 modified CD4^+ ^T-cells, as compared to cells that received the mutant. They also detected Rev M10 until 2 months post-infusion. Though there was increased survival of Rev M10-expressing cells, the overall numbers of transduced cells were low *in vivo *[36].

Trying to improve upon this trial, they conducted another pilot study in which they transduced CD4^+ ^cells with Rev M10, but this time they used retroviral vectors instead of a nonviral vector [37]. They detected Rev M10 for an average of 6 months post-infusion. In addition, cells transduced with Rev M10 survived longer than those transduced with the negative control vector. There were no detectable immune responses to the 'foreign proteins' (Rev M10 or MLV gp70 envelope protein). Though these studies with Rev M10 showed an improved efficacy of gene delivery with a retroviral vector, there was no effect on the patients' viral loads [37].

In 2002, Kang *et al*. published the results of another trial that used a transdominant negative mutant Rev protein (TdRev) [38]. This study had only two subjects. Both were HIV positive and had malignancies. One had leukemia and the other had refractory Hodgkin's lymphoma. Fourteen days prior to receiving gene therapy, the researchers stopped their cyclosporine and HAART medications. Six days prior to gene therapy, the patients received fludarabine-cyclophosphamide containing regimen (non-myeloablative) for five days and then cyclosporine and HAART was restarted. Each patient had an HIV negative sibling who was an HLA-compatible donor for bone marrow cells. The donor underwent blood apheresis followed by isolation of CD34^+ ^cells. The cells were genetically modified by using either TdRev or a control vector encoding human GP91phox. Following transplantation with these modified CD34^+ ^syngeneic cells, the patients developed CMV antigenemia, which was treated. By day 96 post-transplantation, both patients showed 100% transfer of either gene into lymphoid and myeloid lineages. Both patients developed acute graft versus host reaction beyond day 100, which was successfully treated. The patient with Hodgkin's disease died 12 months after transplantation due to relapse of the disease unrelated to any complication of the gene therapy [38]. In a follow-up paper published by the same group, the second patient showed persistence of TdRev at three years post-treatment and continued to be in remission. However, this might be due to the effect of HAART and not TdRev alone [39].

A 2005 study involving the transdominant negative protein approach was focused on the pediatric age group of HIV-positive patients [40]. Two children were enrolled in this study, a nine-year-old and an eight-year-old who were both on HAART. CD34^+ ^bone marrow cells from the participants were transduced with two retroviral vectors, one encoding a "humanized" dominant negative REV protein (huM10) and one encoding an internal control for gene marking (FX) that is not translated. A humanized protein is one in which the codon usage has been optimized for mammalian expression. Following infusion of the modified cells, huM10 and FX could be detected in peripheral blood mononuclear cells (PBMC) for 1–3 months. During a two-year follow-up period, levels of huM10 and FX expression dropped to at or below the limit of detection. In one patient, during a period of non-compliance to HAART regimen, PBMCs containing huM10 reappeared suggesting a selective increase in survival for PBMCs containing huM10 during periods of high viral loads [40].

Using a chimeric receptor approach, Walker *et al*. (2000) investigated the effect of CD4ζ-modified syngeneic T-cells in HIV-positive patients [41]. The study was conducted using sets of twins, one of whom was HIV-positive and the other HIV-negative. The HIV-negative twin acted as the donor for syngeneic T-cells (either CD8^+ ^or CD4^+^), while the HIV-positive twin was the recipient. T cells from the donor were genetically modified *ex vivo *to express CD4ζ and then transfused into the recipient twin. The study was performed in two phases.

In the first phase, 27 patients were enrolled and received one to six cell infusions every eight weeks. Three patients received 10^7 ^modified cells, while the remaining 24 patients were randomly assigned to four groups, receiving either 10^8^, 10^9^, or 10^10 ^modified cells or 10^10 ^unmodified cells. The researchers observed that one of three subjects receiving 10^7 ^cells and four of six subjects receiving 10^8 ^cells showed low levels of CD4ζ expression. In three of these five CD4ζ-positive patients, CD4ζ was no longer detected after one to three days, but in two of them, CD4ζ could be detected for up to 2 and 24 weeks, respectively. In 11 of 12 patients receiving higher doses and multiple infusions of modified CD8^+ ^cells, CD4ζ could be detected for up to 15 to 40 weeks post-infusion [41].

In the second phase of the Walker *et al*. trial, 33 patients were enrolled, 25 from the previous phase and 8 new participants [41]. Of these, three did not receive any cell infusions. Patients received modified CD8^+ ^cell infusions either alone or with IL-2. The group receiving IL-2 with the modified CD8^+ ^cells showed higher levels of persistence of CD4ζ as compared to the group receiving no IL-2 supplementation. In order to test whether the IL2 was substituting for HIV-specific CD4^+ ^T-cell help, 17 of the 30 participants received modified CD4^+ ^cells along with modified CD8^+ ^cells in a second series of infusions. Modified CD8^+ ^and CD4^+ ^cells were detected in the peripheral blood of these 17 patients for at least one year post-infusion, indicating that co-administration of the CD4^+ ^cells may have increased survival of the modified CD8^+ ^cells [41].

In the same year that Walker *et al*. reported their findings, Mitsuyasu and fellow researchers published the results of a similar chimeric receptor study [42]. Patients enrolled in this study were receiving anti-retroviral therapy (ART) and had viral loads between 1,000 and 100,000 copies/ml and CD4^+ ^T cell counts greater than 50 per microliter. Following cell infusions, patients were followed for eight weeks. Eleven patients received CD4ζ-modified T cells along with IL-2, and thirteen patients received CD4ζ-modified T cells alone. In contrast to the previous study by Walker *et al*., administration of IL-2 did not increase the survival of the modified T cells. However, an increase in cell number was observed at eight weeks post-infusion; an average increase of 73 CD4^+ ^cells per microliter was observed in the group receiving IL-2 as compared to the group that did not receive IL-2. They detected CD4ζ in 1 to 3% of the peripheral blood mononuclear cells (PBMCs) at eight weeks and 0.1% at one year post-infusion. CD4ζ-modified T-cells were also isolated from bulk rectal tissue and/or lamina propria lymphocytes in five of five patients at 14 days and two of three patients at one year, showing good tissue distribution. In addition, there was a transient decrease of greater than 0.5 log mean in rectal tissue-associated HIV RNA for at least 14 days [42].

Encouraged by data from this trial, the same group conducted a phase II randomized trial of CD4ζ gene-modified versus unmodified T cells in adult male HIV-positive patients in 2002 [43]. All participants were on combination ART. In 37 patients, there were no measurable viral loads (<50 copies per ml). In three patients, low levels were detected (53, 57 and 65 copies per ml). Only 40 of 42 enrolled patients proceeded to receive the study treatment. The researchers found good expression of CD4ζ for at least 24 weeks in all patients. However, no significant difference was found between patients receiving gene therapy versus the control group when the six following parameters were analyzed: plasma viral load, HIV co-culture on PBMCs, blood HIV DNA, rectal biopsy DNA, rectal biopsy RNA, and blood CD4^+ ^cell count [43].

### RNA-based approaches used in the clinic

A clinical trial involving RNA decoys was conducted in a pediatric population using an RRE decoy to modify CD34^+ ^bone marrow cells. Kohn *et al*. (1999) showed that retroviral mediated CD34^+ ^cell transduction had no significant adverse effects and that leukocytes containing an RRE decoy could be isolated from peripheral blood, even one year post-infusion; however, the numbers of transduced cells were extremely low [44]. Four HIV-positive patients, three teenagers, and one eight-year-old were enrolled in this pilot study. Bone marrow cells positive for CD34 were isolated from these patients and transduced with Moloney murine leukemia (MoMuLV) vector virus carrying the RRE decoy sequences. No change in the HIV viral load was observed among the participants [44].

In 2004, Amado *et al*. demonstrated long-term maintenance of a therapeutic transgene in a phase I clinical trial involving ribozymes [45]. They used MoMLV vector virus transduction of CD34^+ ^HSCs for introduction of a ribozyme targeted to highly conserved regions in the HIV-1 *tat *and *vpr *genes. Ten patients participated in the study and researchers could detect the vector in naïve T cells for up to three years, the last time-point evaluated. There was an average increase of 10 CD4^+ ^T cells per microliter from the beginning of the trial until the third year. In six patients, viral loads decreased by an average of 2.25 logs. Three patients had undetectable viral loads. One patient showed an increase of one log. The researchers found no correlation between the changes in viremia or CD4^+ ^T cell counts with vector expression or detection in any cell type. However, during this trial, all patients were on ART, and the researchers attribute the changes in viral load as well as the cell numbers to individual viral susceptibility to ART [45].

More recently in 2005, Macpherson *et al*. published the results of a phase I clinical trial on ribozymes involving identical twins with discordant HIV status [46]. Again, one twin acted as the donor (HIV-negative) and the other was the recipient (HIV-positive) of genetically engineered CD4^+ ^T cells expressing a ribozyme. Specifically, the cells were transduced with an anti-HIV-1 *tat *ribozyme (RRz2) and a control LNL6 retroviral vector (for cell marking). Patients were followed initially for 24 weeks and then at regular intervals over a 4-year period. PBMCs containing both RRz2 and LNL6 were detected consistently. However, the effect of this therapy on HIV-1 viral load or the CD4 count was not specifically addressed.

Lastly, Morgan *et al*. (2005) published data from a clinical trial involving anti-sense RNA in conjunction with a trans-dominant negative protein [47]. This study employed 10 pairs of twins. Like the earlier study involving twins with discordant HIV status, one twin served as the donor (HIV-negative) and the second twin as the recipient (HIV-positive). Lymphocytes from the donors were transduced to express a control gene (*neo *gene) or anti-HIV gene(s); a transdominant mutant Rev protein (TdRev) was used alone or with an anti-sense element directed against the HIV-1 TAR sequence on the same construct. Polymerase chain reaction demonstrated increased survival of modified lymphocytes in the initial weeks post-infusion in 9 of 10 recipients. In six of six recipients followed for approximately two years, T cells containing anti-HIV genes could be consistently detected and there was preferential survival of modified cells in one patient during a period of high HIV load [47].

## Conclusion

Early promising results on the treatment of adenosine deaminase-severe combined immunodeficiency (ADA-SCID) by using gene therapy in the early 1990s [48] led the scientific community to apply the same principle to a host of other diseases, including HIV/AIDS. *In vitro *studies quickly demonstrated the feasibility of such approaches and preclinical and clinical trials were started later in the same decade. However, it was soon realized that a cure for HIV was far from easy. As many studies demonstrated, there were no serious adverse effects of the therapy, but neither was there any decrease of patients' viral loads. There was also the problem of transduction efficiency, and transduced cells had only transient expression of the transgene. Moreover, there was no consensus on target genes and methodology.

Despite some discouraging results from clinical trials, gene therapy for HIV is still a very promising approach. Scientists are already overcoming the problems of insufficient gene transduction by using novel constructs [49] or by switching to lentiviral-mediated transduction [50,51]. The first clinical trial using lentiviral vectors in HIV-positive patients began in 2001, and results will be forthcoming [52]. The recent clinical studies by Podsakoff *et al*. and Morgan *et al*. in 2005 support the hypothesis that anti-HIV genes confer a survival advantage to modified lymphocytes [40,47], especially under conditions of high HIV titers, thus offering a potential benefit to infected individuals.

Other promising strategies for the near future include the use of siRNAs and fusion proteins to deliver these molecules [53-55]. Early HIV regulatory genes like *tat *and *rev *could be susceptible targets for siRNA because genes encoding late structural proteins are dependent on Tat and Rev protein expression [56]. Scherer *et al*. showed that *tat*- and *rev*-specific siRNAs were more effective at inhibiting HIV-1 replication than multiple siRNAs targeting *env *[57]. Other research groups have demonstrated success *in vitro *by targeting Gag or HIV receptors with siRNA [58,59]. Song *et al*. (2005) took advantage of the nucleic acid binding properties of protamine and fused it to the heavy chain Fab fragment of an anti-HIV Env antibody to deliver siRNA to HIV-infected cells [54].

According to von Laer *et al*. (2006), antiviral genes that inhibit the processes of reverse transcription and integration potentially offer significant therapeutic benefit [60]. They based their prediction on mathematical models, which analyzed the effects of genetically modified T cells on viral replication and T cell kinetics. This strategy could include targeting cellular factors involved in these enzymatic processes. Developing siRNAs that target reverse transcriptase and integrase genes themselves have potential to be effective anti-HIV therapies. Although these genes code for late protein products, inhibition of these genes could create defective virus particles incapable of initiating subsequent replication cycles.

While the initial achievements using siRNA against HIV have employed transient expression systems, stable systems have been reported and offer hope for long-term efficacy [30,53,61-65]. The specificity of siRNAs reduces the potential side effects of gene therapy but, on the other hand, it increases the possibility of making a virus partially or completely resistant to siRNA with the slightest mutation [66-69]. The strategy of simultaneously targeting several conserved regions of HIV using multiple different siRNAs could potentially overcome this problem of viral escape.

In conclusion, gene therapy is a very attractive method for treating HIV-positive patients. The approaches undertaken so far have yielded encouraging results from a safety efficacy standpoint. Future efforts should focus on improving transduction efficiencies and long-term expression and on optimizing cellular targets in order to achieve the desired therapeutic benefits, which include decreasing viral loads, increasing or sustaining high CD4^+ ^T cell counts, and improving immune function in HIV-infected individuals.

## Competing interests

The author(s) declare that they have no competing interests.

## Authors' contributions

JGM and DPW produced the manuscript together. Both authors read and approved the final manuscript.
